# Destroying Nerves and Filling Cavities

**Published:** 1862-07

**Authors:** W. H. Shadoan


### DESTROYING NERVES AND FILLING CAVITIES.

BY W. H. SHADOAN.

In several numbers of the Register I have, in as plain and simple a manner as possible, attempted to give a description of the process of destroying the nerves of the teeth, and the success obtained. I know that a great many dentists, some of whom are said to be *good* practitioners, deny the fact of the permanency of a job, after the tooth has ached; while other practitioners, of equal attainments, assert in the most positive terms that it has been done, and they (the teeth thus treated) made as permanent as any of the other teeth.

In the thirteenth volume of the Register, page 117, I gave an idea of my process of the treatment of such cases, and in volume fifteen, under the head of "Alveolar Abscess—Treatment and Cure," you will find anothor case, which, if taken as proof in the present case, will go far towards proving that if a tooth aches, it is not sufficient reason that it must be sacrificed on the altar of some bungler's "Cant hook."

What is a dentist, or what are his duties? The dentist most assuredly ought to know more than simply to "pull a tooth," or plug one, or make a substitute. If I understand the term Doctor of Dental Surgery, it means a man skilled in the art of curing the disease of the mouth, coming in what shape it may. If a tooth is diseased at the root, why not cure it as well as the physician cure his patient of periostitis? The fact of it being a tooth does not necessarily follow that it must be extracted if it gets sick, or if the nerve becomes diseased, that the tooth must be lost, no matter where it is situated, whether as a grinder or a cutter, or whether the decay is slight or extended, so as to leave nothing but a shell, I say I deny it. I have cured teeth of almost all manner of disease, and under favorable circumstances, it can always be done.

I have not taken up with this "hobby," as some would call it, merely to have something to boast of by any means. On the contrary, I have taken up with it from an honest belief that all can be done that is claimed. Who would think of amputating an arm because there is a boil, or a leg because there is a supposed white swelling? Employ undoubted skill, and save the leg if possible; if after all the means brought to bear upon it fail, there is a "dernier resort"—amputate it. So with the tooth, if after the tooth has been skillfully treated, and it can not be cured, then, and not till then, you may remove it.

I have within the last three months had at least a dozen teeth to treat, that were pronounced incurable by others. Speaking of others failing to treat and cure disease of the teeth, reminds me of two brothers—both graduates of "Ohio Dental College." (I give their place of education, not as a criticism on the institution, but to show how they fail to try to do what they were instructed to do in the valedictory address, "Do all you can.") They say that after a tooth has once ached, that it is no use to fill it. That it will be sure to give trouble, etc. I was in their place of business (I will not call it an office) some time since. While there, a gentleman came in, and asked them to fill a tooth for him, at the same time remarking it had ached. He refused to fill it. The man has since called on me for the same operation. These same "*graduates*" have not taken a dental periodical for seven years. They discontinued the Register at that time. Now, I don't take the Register as my Bible, but for my part I don't see how a dentist practicing his profession can get along without it.

My process is about the same that it was five years ago. I destroy the nerve with cobalt and creosote, sometimes with arsenous acid. Treat with solution of chlorate of zinc, creosote, and sometimes Teft's anæsthetic. After the parts are free from soreness, I fill fangs with gold foil and creosote. I most always saturate a small pledget of cotton with creosote, and put it up in the fang as far as I can. The creosote forms an insoluble compound with any remaining particles of nerve or dentine, so that it matters not in as small a place as that, whether all the impurities are removed or not.* After the fangs have been filled from one to six weeks, owing to the extent of the disease, if there are no signs of returning pain, I fill the balance of the cavity, and will say that I have not lost one case in twenty. In some cases, I have filled the crown cavity with os-artificial, which I prefer in cases where the filling is not exposed to the food in eating; in that event, I prefer gold—the former being more liable to wear or wash out. My experience is, that the os-artificial is a very poor conductor of heat, which I think in fang filling is a very important point. I do not put it in the nerve cavity, from the fact that in some teeth it softens the dentine in the fangs, and in some cases it produces inflammation for a short time, and for these I prefer other and more reliable materials. As to crown cavities, they are surrounded with more dense bone than the fangs. It is drier, and consequently is not so liable to be affected. The reader might say, If you use it in one case, why not in all? I only use it in cases where the result would be more uncertain with gold. If I fail with the former, the loss to the patient is not so great as in gold. I filled a tooth on the first of November last for a gentleman of this place, as described above. A few days ago we were talking about it, and he said that he had not had the slightest trouble since it was filled. I have others of the same kind, but wil not refer to them. They are all in good condition.

---

* We think it important to remove every particle of loose tooth-bone or other substance from the canal, leaving the clear clean bone. It is best to urge the most perfect practice in every particular. More is sometimes taken from such admisions than is intended.—Ed.

